# Quantitative susceptibility mapping to evaluate the early stage of Alzheimer's disease^☆^

**DOI:** 10.1016/j.nicl.2017.08.019

**Published:** 2017-08-24

**Authors:** Hyug-Gi Kim, Soonchan Park, Hak Young Rhee, Kyung Mi Lee, Chang-Woo Ryu, Sun Jung Rhee, Soo Yeol Lee, Yi Wang, Geon-Ho Jahng

**Affiliations:** a Department of Biomedical Engineering, Graduate School, Kyung Hee University, 1732, Deogyeong-daero, Giheung-gu, Yongin-si, Gyeonggi-do 446-701, Republic of Korea; b Department of Radiology, Kyung Hee University Hospital at Gangdong, College of Medicine, Kyung Hee University, 892 Dongnam-ro, Gangdong-Gu, Seoul 05278, Republic of Korea; c Department of Neurology, Kyung Hee University Hospital at Gangdong, College of Medicine, Kyung Hee University, 892 Dongnam-ro, Gangdong-Gu, Seoul 05278, Republic of Korea; d Department of Radiology, Kyung Hee University Hospital, College of Medicine, Kyung Hee University, 23 Kyungheedae-ro, Dongdaemun-gu, Seoul 02447, Republic of Korea; e Department of Biomedical Engineering and Radiology, Cornell University, 515 E 71st Street, Suite 102, New York, NY 10021, USA

**Keywords:** Alzheimer's disease (AD), Mild cognitive impairment (MCI), Quantitative susceptibility mapping (QSM), Gray matter volume

## Abstract

The objective of this study was to evaluate susceptibility changes caused by iron accumulation in cognitive normal (CN) elderly, those with amnestic mild cognitive impairment (aMCI), and those with early state AD, and to compare the findings with gray matter volume (GMV) changes caused by neuronal loss. The participants included 19 elderly CN, 19 aMCI, and 19 AD subjects. The voxel-based quantitative susceptibility map (QSM) and GMV in the brain were calculated and the differences of those insides were compared among the three groups. The differences of the QSM data and GMVs among the three groups were investigated by voxel-based and region of interest (ROI)-based comparisons using a one-way analysis of covariance (ANCOVA) test with the gender and age as covariates. Finally, a receiver-operating-characteristic (ROC) curve analysis was performed. The voxel-based results showed that QSM demonstrated more areas with significant difference between the CN and AD groups compared to GMV. GMVs were decreased, but QSM values were increased in aMCI and AD groups compared with the CN group. QSM better differentiated aMCI from CN than GMV in the precuneus and allocortex regions. In the accumulation regions of iron and amyloid β, QSM can be used to differentiate between CN and aMCI groups, indicating a useful an auxiliary imaging for early diagnosis of AD.

## Highlights

• The susceptibility difference was more sensitive than GMV change in known regions of iron and the amyloid β accumulations.
• QSM values were increased in patients compared with normal elderly and better differentiated aMCI from CN than GMV values.
• The QSM technology can be used as an auxiliary imaging for early diagnosis of AD.

## 1 Introduction

Alzheimer's disease (AD) is the most common progressive and complex age-related neurodegenerative disorder, leading to cognitive decline (Albertini et al., 2012) and memory loss (Jahn, 2013). Mild cognitive impairment (MCI) is a high-risk condition for the development of clinically probable AD or other neurological conditions (Petersen et al., 2001). The onset of AD is commonly preceded by an interim phase known as amnestic MCI (aMCI) (Shah et al., 2000). The assessment of aMCI is beneficial in terms of early AD intervention and perhaps of AD prevention (Sherwin, 2000). The hallmarks of AD may manifest as alterations of susceptibility in a certain area in the brain, caused by associated iron overload (Haacke et al., 2005), and demyelination of white matter (Carmeli et al., 2014). Loss of myelin causes the susceptibility of white matter to increase, approaching that of gray matter (Lee et al., 2012). Both demyelination and iron deposition increase local tissue susceptibility (Liu et al., 2015). Elevated levels of brain iron have been suggested to be a risk factor for AD (Duce et al., 2010, Ayton et al., 2015).

Due to the relevance of susceptibility properties to physiological changes, a number of studies have been performed to observe susceptibility changes using MRI, especially to detect increase iron contents by T2* map (Saarlas et al., 2006) and by susceptibility weighted images (SWI) (Haacke et al., 2009). However, those MRI techniques are not useful for measuring because they suffer from blooming artifacts (Li et al., 2012) and cannot quantify tissue susceptibility (Schenck and Zimmerman, 2004). Quantitative susceptibility mapping (QSM) (de Rochefort et al., 2010, Wang and Liu, 2015) is a MRI technique that enables quantification of susceptibility-changing materials (Schweser et al., 2012).

Magnetic susceptibility refers to a physical property of a material that is useful for chemical identification and quantification of specific materials, including iron, calcium, and contrast agents. Most biological substances, such as calcium and white matter myelin, are slightly diamagnetic, meaning that they induce small negative susceptibility changes. However, iron stored in ferritin, hemosiderin, and neuromelanin in brain tissue and iron embedded in deoxyhemoglobin in venous blood is highly paramagnetic and causes strong magnetic field (Bilgic et al., 2012).

QSM can be used to study iron involvement in AD, but few papers have reported the difference of the QSM values between AD and controls in a very limited subject population (Acosta-Cabronero et al., 2013, Moon et al., 2016). Although previous studies have examined the brain iron concentration in normal aging and reported different susceptibility values between AD and controls using R2* and SWI, no studies have examined the susceptibility changes in the early state of AD using QSM. Furthermore, in vivo iron distributions in brains of patients with early state AD are still unknown. The objective of this study was to systematically investigate the susceptibility changes in subjects of CN, aMCI, and early stage AD using voxel-based analysis and region of interest (ROI)-based analysis, and to compare the data to gray matter volume (GMV) changes caused by neuronal losses in the subjects.

## 2 Materials and methods

### 2.1 Subjects

Our institutional review board approved this prospective study and informed consent was obtained from all participants. All participants provided a detailed medical history and underwent a neurologic examination, standard neuropsychological testing, and MRI scan. Cognitive functions were assessed using the full Seoul Neuropsychological Screening Battery (SNSB), which is a standardized neuropsychological battery in Korea that comprises tests for attention, visuospatial function, verbal and visual memory functions, language-related function, and frontal executive function (Han et al., 2010). A neuroradiologist with 15 years of imaging experience evaluated brain MR images for each subject to determine if study subjects had any evidence of prior cortical infarctions or other space-occupying lesions.

Based on the results of SNSB examination and MRI findings, CN were selected from healthy volunteers who did not have a medical history of neurological disease, who showed normal results on detailed cognitive testing scores that were within 1 standard deviation (SD) adjusted for age, gender, and education according to the Korean normative database, and who also had a normal brain MRI. Patients with mild and probable AD were included, and these patients were defined as those with the Clinical Dementia Rating (CDR) scores of 0.5, 1, or 2, according to the National Institute of Neurological and Communicative Disorders and Stroke-Alzheimer Disease and Related Disorders Association (NINCDS/ADRDA) criteria (McKhann et al., 1984). aMCI was also included, according to the Petersen criteria (Petersen et al., 1999). Patients with vascular dementia were excluded in this study.

We recruited a total of 69 subjects from the neurologic clinic center in our institute. After reviewing clinical database as well as MRI, 12 subjects were excluded because of streaking artifacts in QSM data (*n* = 5), wrong scanning of the T1-weighted image (*n* = 1), brain hemorrhage (*n* = 1), abnormal neuropsychological findings (*n* = 2), and withdraw prior to finishing the SNSB examination and MRI scan (*n* = 3). The remaining 57 participants included 19 elderly CN, 19 aMCI, and 19 mild and probable AD. Table 1 summarizes the demographic characteristics and results of neuropsychological tests of the subjects.

**Table 1:** Demographic data and the neuropsychologic test results of cognitive normal (CN), amnestic mild cognitive impairment (aMCI), and Alzheimer's disease (AD).

	CN	aMCI	AD
Subjects	19	19	19
Age^a^	65.37 ± 6.29	65.95 ± 6.75	69.79 ± 10.27
Gender^b^			
Male	3	9	2
Female	16	10	17
K-MMSE^c^	28.16 ± 1.89	27.63 ± 2.11	17.37 ± 3.42
CDR	0.0 (0.0–0.5)	0.5 (0.0–0.5)	1.0 (1.0–2.0)

Abbreviations: K-MMSE, Korean Mini-Mental State Examination Score; CDR, Clinical Dementia Rating.

Note. The data of age and K-MMSE scores are presented as the mean ± standard deviation, but those of CDR scores are presented as the median (range) value.

a Age in years significantly differed between the CN and other groups (*p* < 0.05), but there was no significant difference between the aMCI and AD group (*p* > 0.05).

b Genders are significantly different between CN and aMCI (*p* = 0.0013), but not between aMCI and AD (*p* = 0.0665) and also between CN and AD (*p* = 0.0665).

c K-MMSE scores were significantly different between the CN and other groups (*p* < 0.05) and between the aMCI and AD groups (*p* < 0.05).

### 2.2 Image acquisition

MRI scans were acquired in each subject using a 3 T MR system (Achieva, Philips Medical Systems, Best, The Netherlands) equipped with an eight-channel sensitivity encoding (SENSE) head coil. To evaluate the susceptibility changes in the brain, a 3D fast field-echo (FFE) sequence was run with seven echoes to obtain magnitude and phase images with the following parameters: repetition time (TR) = 43 ms, first echo time (TE)/ΔTE/final TE = 3.4/6.0/39 ms, flip angle (FA) = 20°, field-of-view (FOV) = 220 × 198 mm^2^, acquisition voxel size = 0.68 × 0.68 × 2.20 mm^3^, and voxel size = 0.63 × 0.63 × 2.00 mm^3^. To evaluate the GMV changes, sagittal structural 3D T1-weighted (T1W) images were acquired with the magnetization-prepared rapid acquisition of gradient echo (MPRAGE) sequence with the following parameters: TR = 8.1 ms, TE = 3.7 ms, FA = 8°, FOV = 236 × 236 mm^2^, acquisition voxel size = 1 × 1 × 1 mm^3^, and voxel size = 1 × 1 × 1 mm^3^. In addition, T2 W turbo-spin-echo and fluid-attenuated inversion recovery (FLAIR) images were acquired to exam any malformation of the brain.

### 2.3 Processing of 3D FFE images to obtain QSM

To generate QSM, the acquired magnitude and phase images from the 3D FFE sequence were further processed by implementing the Morphology Enabled Dipole Inversion (MEDI) method (Liu et al., 2012a, Liu et al., 2011b, Liu et al., 2012b) with the following steps: estimation of the total field (Wei Xu, 1999), generation of the tissue field to remove the background field using the projection onto dipole fields (PDF) (Liu et al., 2011a), process of the field-to-susceptibility inversion to solve the ill-posed inverse problem, and generation of QSM map.

### 2.4 Post-processing of QSM and gray matter volume from 3D T1 W image

The following post-processing steps were performed using a Statistical Parametric Mapping Version 8 (SPM8) program (Wellcome Department of Imaging Neuroscience, University College, London, UK). First, the 3D T1W image was co-registered to a magnitude image acquired from the 3D FFE sequence of the same subject. All 3D T1W images were then segmented into gray matter (GM), white matter (WM), and cerebrospinal fluid (CSF). Using these segmented tissues, the study-specific brain template was created using a Diffeomorphic Anatomical Registration Through Exponentiated Lie Algebra (DARTEL) tool (Ashburner, 2007). The segmented GM of each subject was spatially normalized into the created template. The spatially normalized GMV was smoothed using the Gaussian kernel of 8 × 8 × 8 mm full width at half-maximum (FWHM).

Second, the QSM map of each individual subject was spatially normalized into the created brain template on the MNI space using the normalized parameters of the 3D T1W image. After the spatial normalization, the QSM maps were processed to minimize contamination of the CSF effects, which was not a concern for the study, by using the following steps. Voxels containing > 80% GM and WM obtained from the segmented 3D T1W images were selected and were masked out otherwise. The mean QSM value in each voxel was subtracted by the internal standard reference values which were estimated by the average of the QSM values in the bilateral posterior ventricular region for each subject (Acosta-Cabronero et al., 2013), since the values calculated from MEDI are relative with an unknown offset. Finally, QSM maps were smoothed by using the Gaussian kernel of 8 × 8 × 8 mm FWHM.

### 2.5 Definition of region of interest (ROI)

ROIs were defined two different ways. The first involved the well-known regions for rich iron contents of the brain, reported in two previous studies (Connor et al., 1992, Haacke et al., 2005). Therefore, we set knowledge-based ROIs, which were the amygdala, globus pallidus, hippocampus, precuneus, pulvinar, putamen, and thalamus. Second, several amyloid positron emission tomography (PET) studies showed amyloid accumulations in the AD brain, which may be related to increased iron accumulation (Vandenberghe et al., 2013). Therefore, we set amyloid β accumulation-based ROIs, which were the neocortex (Brodmann area; BA #24, 25, and 30), allocortex (BA #4), entorhinal cortex (BA #28, 34), anterior cingulate cortex (ACC) (BA #24, 32, 33), and posterior cingulate cortex (PCC) (BA #23, 31) (Vandenberghe et al., 2013). These areas were automatically traced on the brain atlas space using the WFUPickAtlas software (fmri.wfubmc.edu/software/pickatlas). The mean values of GMV and QSM were obtained from the 13 defined ROIs using Marsbar software (Matthew Brett, marsbar.sourceforge.net).

### 2.6 Statistical analyses

#### 2.6.1 Demographic characteristics and results of neuropsychological tests

Demographical data and clinical outcome scores were compared among the three groups. Age and Mini-Mental State Examination (MMSE) scores were not normally distributed (*p* < 0.005 by Kolmogorov-Smirnov test). Hence, age and MMSE scores were tested using the Kruskal-Wallis test. If we found any significant differences among the groups, then we performed a post hoc test for pairwise comparison of subgroups according to the Conover method (Conover, 1999). Gender was tested using the Chi-squared test.

#### 2.6.2 Voxel-based analysis of GMV and QSM

To compare GMV and QSM among the three groups, voxel-wise, one-way analysis of covariance (ANCOVA) test was used with subject's age and gender as covariates. A significance level of *p* = 0.05 was applied with correction for multiple comparisons using the family-wise error (FWE) method and clusters with at least 10 contiguous voxels.

#### 2.6.3 ROI-based analyses of GMV and QSM

Because ROI for GMVs and QSM were normally distributed (*p* > 0.05 by the Kolmogorov-Smirnov test), we used the following parametric test. Because we had several different ROIs within each subject and the three subject groups, two-factor repeated measures ANOVA was used as the first factor for the three subject groups. The second factor was ROIs. In this analysis, we evaluated the between-subject effects and the within-subject effects. After the two-factor analysis, we reanalyzed GMVs and QSM using the one-factor repeated measures ANOVA to evaluate differences of GMVs and QSM among ROIs. Furthermore, to evaluate group differences of GMVs and QSM among the three subject groups for each ROI, ANCOVA was used with age and gender as covariates. The post-hoc test was performed by using the pairwise comparisons of those values between subject groups with Bonferroni corrected *p* = 0.05.

#### 2.6.4 Receiver operating characteristic (ROC) curve analyses

ROC curve analysis was performed to demonstrate sensitivity and specificity of GMVs and QSM to differentiate among the subject groups for each ROI. A significance level of Bonferroni corrected *p* = 0.05 was also applied for this analysis. MedCalc statistical software (www.medcalc.org, Ostend, Belgium) was used to analyze the ROI.

## 3 Results

### 3.1 Subject characteristics

The demographic data of the subjects are summarized in Table 1. Ages were significantly different among the three groups (*p* = 0.0125). Ages were significantly different between the CN and other groups (*p* < 0.05) and between the aMCI and AD (*p* < 0.05). Genders were significantly different between the CN and aMCI (*p* = 0.0013), but not between the aMCI and AD (*p* = 0.0665) and also between the CN and AD (*p* = 0.0665). The MMSE scores were significantly different between the groups (*p* < 0.0001). MMSE scores were significantly different between the CN and other groups (*p* < 0.05) and between the aMCI and AD (*p* < 0.05).

**Fig. 1:**
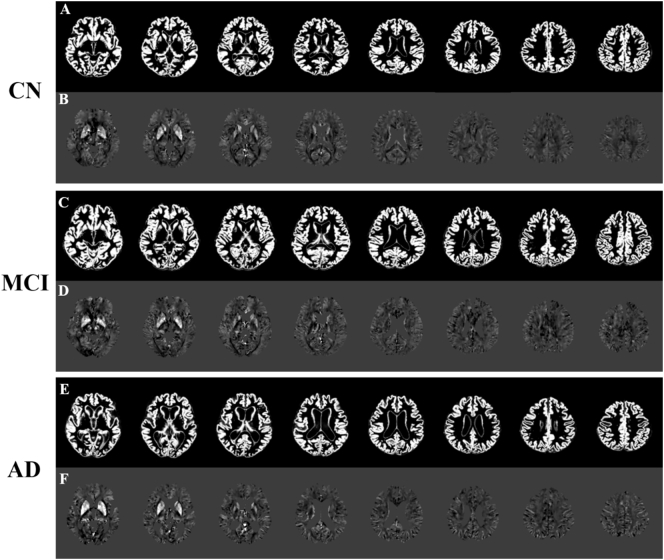
Representative maps of gray matter volumes (A, C, and E) and the segmented tissue components of QSM maps (B, D, and F). CN, cognitive normal; aMCI, amnestic mild cognitive impairment; AD, Alzheimer's disease.

Fig. 1 displays representative images of GMVs (Fig. 1A, C, and E) and the corresponding QSM (Fig. 1B, D, and F) for the CN (Fig. 1A and B), aMCI (Fig. 1C and D) and AD (Fig. 1E and F). QSM maps are shown with voxels combining > 80% of GM and WM.

### 3.2 Voxel-based analysis

**Fig. 2:**
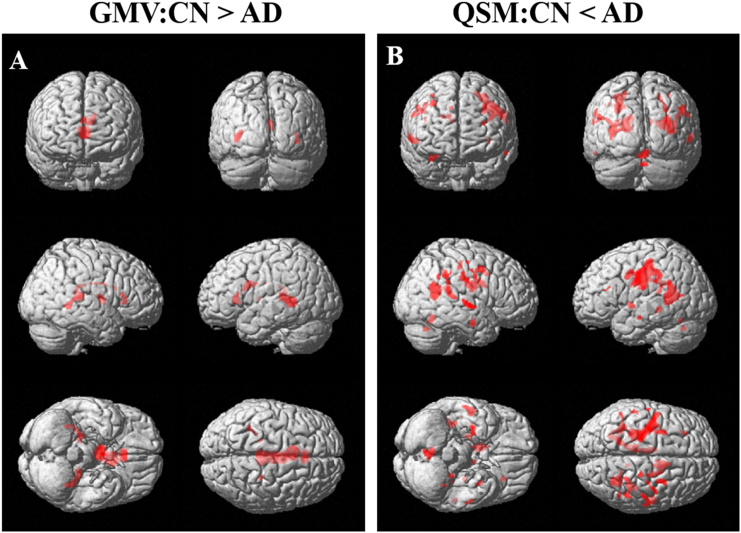
Results of the voxel-based analysis of gray matter volume (GMV, A) and quantitative susceptibility mapping (QSM, B) values between the cognitive normal (CN) and Alzheimer's disease (AD) subjects. GMV (A) obtained from a 3D T1-weighted imaging was higher in CN than that in AD, but QSM value (B) was higher in AD than that in CN. There were no significant differences for both GMVs and QSM values between other subject groups.

The results of voxel-based comparisons of GMV and QSM among the three groups are summarized in Fig. 2 and Table 2. QSM and GMV were significantly different between the CN and AD, but no significant difference between other groups. Increased QSM values in AD compared with CN were found mainly at the left precentral gyrus, left postcentral gyrus, right caudate, right cingulate gyrus, right parahippocampal gyrus, hippocampus, amygdala, caudate, and insula. Decreased GMV in AD compared with CN was found at the left and right thalamus, and the left anterior cingulate, hippocampus, caudate, and cingulate gyrus.

**Table 2:** Areas showing decreased gray matter volumes (GMVs) or increased QSM values in patients with Alzheimer's disease (AD) compared with cognitively normal (CN) elderly subjects.

Group analysis	Cluster size	Cluster location	BA	Talairach coordinates			z score
GMV	160	Lt. hippocampus		− 32.62	− 44.63	1.3	5.73
AD < CN	148	Lt. thalamus		− 1.17	− 4.68	14.62	5.65
	86	Rt. thalamus	Pulvinar	19.15	− 30.69	10.7	5.58
		Rt. hippocampus		32.16	− 43.1	2.54	4.92
		Rt. caudate tail		28.41	− 38.02	8.36	4.77
	440	Rt. thalamus	Pulvinar	6.11	− 34.87	15.49	5.38
		Lt. cingulate gyrus in limbic lobe	23	− 3.19	− 16.89	24.24	5.26
	121	Lt. anterior cingulate in limbic lobe	24	− 1.01	22.11	9.96	5.17
QSM	1833	Lt. precentral gyrus in frontal lobe	6	− 43.1	− 12.98	33.85	5.21
AD > CN		Lt. postcentral gyrus in parietal lobe	3, 43	− 29.33	− 23.91	39.35	5.08
	53	Rt. precentral gyrus in frontal lobe	4, 6	40.2	− 9.69	35.57	4.57
	159	Rt. superior temporal gyrus in temporal lobe	22, 39	50.75	− 10.42	4.15	5.17
	87	Rt. postcentral gyrus in parietal lobe	2	47.55	− 23.79	35.26	5.16
	83	Rt. amygdala		31.62	− 8.32	− 16.7	5.06
	725	Lt. posterior cingulate in limbic lobe	23, 30	− 9.76	− 32.2	27.19	5.02
		Lt. hippocampus		− 33.62	− 46.08	6.55	4.86
	276	Rt. transverse temporal gyrus in temporal lobe	41	34.8	− 38.68	14.72	4.87
		Rt. supramarginal gyrus in Parietal lobe	40	44.68	− 46.39	35.77	4.86
	457	Rt. parahippocampal gyrus in limbic lobe	30	29.25	− 53.28	10.53	4.86
		Rt. posterior cingulate in limbic lobe	29, 30, 31	23.63	− 50.98	16.06	4.84
	20	Lt. inferior parietal lobule in parietal lobe	40	− 54.23	− 35.02	28.87	4.85
	110	Rt. middle frontal gyrus in frontal lobe	6	29.01	− 1.1	44.3	4.68
	61	Rt. cingulate gyrus in limbic lobe	24, 31	26.18	− 44.43	35.64	4.59
	19	Rt. insula	13	34.84	− 13.68	18.88	4.43
	36	Lt. insula	13	− 30.92	− 36.63	15.6	4.4
		Lt. caudate tail		− 25.46	− 40.06	21.67	4.32
	332	Rt. caudate body		18.12	− 11.32	24.23	5.18
	157	Rt. culmen in anterior lobe		3.67	− 56.12	− 17.19	4.96

Abbreviations: BA, Broadmann area; Lt., left; Rt., right.

Note. For both QSM and GMV data, there were significant differences between the CN and AD groups, but no significant difference was evident between the other groups.

### 3.3 ROI-based analyses

**Fig. 3:**
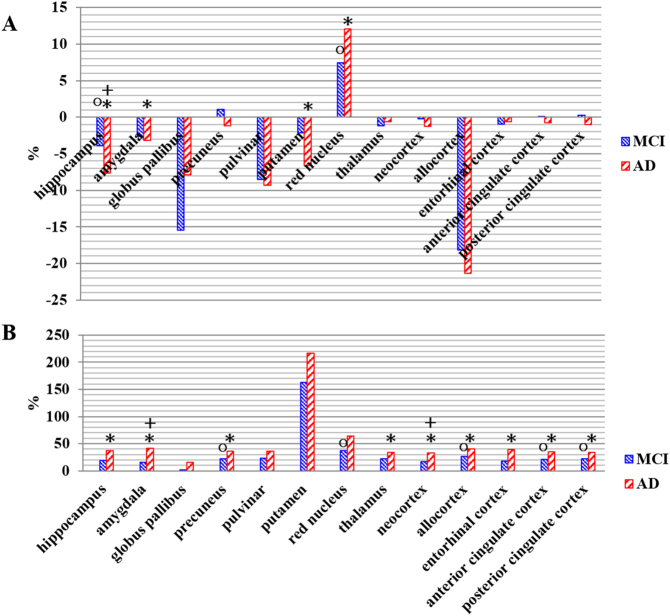
Mean values of percentage changes of gray matter volumes (GMVs: A) and QSM values (B) in the specific regions of interest on the amnestic mild cognitive impairment (aMCI) and Alzheimer's disease (AD) against the cognitive normal (CN) subjects. The result of statistically significant differences of GMV and QSM are indicated by circle (○) for the comparison between CN and aMCI groups, of plus (+) for the comparison between aMCI and AD group, and of asterisk (*) for the comparison between CN and AD group. GMV was lower in aMCI and AD than that in CN, but QSM value was higher in aMCI and AD than in CN.

Fig. 3 shows the percent changes of GMV (Fig. 3A) and QSM (Fig. 3B) values for aMCI and AD compared to CN subjects for each ROI. GMV were significantly different between-subject groups (DF = 2, F = 13.57, *p* < 0.001) and within-subject group (DF = 12, F = 5446.36, *p* < 0.0001). QSM were significantly different between-subject groups (DF = 2, F = 5.63, *p* = 0.006) and within-subject group (DF = 12, F = 452.87, *p* < 0.001). These results indicated that GMV and QSM values were significantly different among the 13 ROIs as well as were significantly different among the three groups. We describe the results of the post-hoc test of GMV and QSM values below.

#### 3.3.1 GMV

Compared to the CN, GMV in the aMCI was significantly decreased at the hippocampus (*p* = 0.0208). GMV in the AD was significantly decreased at hippocampus (*p* < 0.0001), amygdala (*p* = 0.0394), and putamen (*p* = 0.0077). Compared to the aMCI, GMV in the AD was significantly decreased only at the hippocampus (*p* = 0.0209) (Table 3).

**Table 3:** Mean values for GMVs and QSM values obtained from the specific region-of-interests (ROIs) defined in the seven well-known iron accumulation regions and the five amyloid β accumulation regions in the subjects with Alzheimer's disease (AD), amnestic mild cognitive impairment (aMCI), and cognitively normal (CN) elderly.

	ROI		Group (mean ± std)			ANCOVA	
			CN	aMCI	AD	F	*p*
Iron accumulation regions	Hippocampus	GMV^○+^*	0.862 ± 0.008	0.828 ± 0.009	0.796 ± 0.008	14.3760	<* 0.001*
		QSM*	− 32.943 ± 2.099	− 26.550 ± 2.008	− 20.649 ± 2.052	8.1590	*0.0010*
	Amygdala	GMV*	0.738 ± 0.006	0.718 ± 0.006	0.714 ± 0.006	3.7240	*0.0310*
		QSM^+^*	− 32.889 ± 2.345	− 27.567 ± 2.243	− 19.073 ± 2.293	8.5250	*0.0010*
	Globus pallibus	GMV	0.153 ± 0.011	0.129 ± 0.010	0.141 ± 0.011	1.0830	0.3460
		QSM	38.040 ± 4.672	38.721 ± 4.468	44.097 ± 4.568	0.5070	0.6060
	Precuneus	GMV	0.451 ± 0.004	0.456 ± 0.004	0.445 ± 0.004	1.5750	0.2170
		QSMo*	− 31.871 ± 1.859	− 24.686 ± 1.778	− 20.496 ± 1.817	9.0430	<* 0.001*
	Pulvinar	GMV	0.382 ± 0.002	0.350 ± 0.002	0.347 ± 0.002	1.2180	0.3040
		QSM*	− 24.976 ± 2.277	− 19.253 ± 2.177	− 16.041 ± 2.226	3.7290	*0.0310*
	Putamen	GMV*	0.313 ± 0.004	0.306 ± 0.004	0.293 ± 0.004	5.3310	*0.0080*
		QSM	− 3.238 ± 2.047	2.047 ± 2.789	3.770 ± 2.851	1.4770	0.2380
	Thalamus	GMV	0.259 ± 0.001	0.256 ± 0.001	0.258 ± 0.001	0.7180	0.4930
		QSM*	− 30.153 ± 2.129	− 23.543 ± 2.036	− 19.915 ± 2.081	5.6100	*0.0060*
Amyloid β accumulation regions	Neocortex	GMV*	0.676 ± 0.002	0.675 ± 0.002	0.668 ± 0.002	3.4750	*0.0380*
		QSM^+^*	− 38.929 ± 1.952	− 32.511 ± 1.866	− 25.985 ± 1.908	10.4830	<* 0.001*
	Allocortex	GMV	0.271 ± 0.004	0.222 ± 0.004	0.2138 ± 0.004	0.9170	0.4060
		QSM^○^*	− 30.967 ± 1.893	− 22.907 ± 1.811	− 18.403 ± 1.851	10.6540	<* 0.001*
	Entorhinal cortex	GMV	0.629 ± 0.005	0.623 ± 0.005	0.625 ± 0.005	0.2650	0.7680
		QSM*	− 30.514 ± 2.085	− 25.111 ± 1.993	− 18.431 ± 2.038	8.0790	*0.0010*
	ACC	GMV	0.621 ± 0.002	0.622 ± 0.002	0.616 ± 0.002	1.1780	0.3160
		QSM^○^*	− 33.291 ± 1.871	− 26.429 ± 1.789	− 21.478 ± 1.829	9.5200	<* 0.001*
	PCC	GMV	0.588 ± 0.002	0.589 ± 0.002	0.582 ± 0.002	2.0780	0.1350
		QSM^○^*	− 32.540 ± 1.960	− 25.173 ± 1.875	− 21.368 ± 1.916	7.9170	*0.0010*

Abbreviations: GMV, gray matter volume; QSM, quantitative susceptibility mapping; ppb, parts per billion; std., standard deviation; ANCOVA, analysis of covariance; ACC, anterior cingulate cortex; PCC, posterior cingulate cortex; F, F-test; *p*, *p*-value.

Note. The result of statistically significant differences of GMV and QSM are indicated with the symbols of circle (○) for the comparison between CN and aMCI groups, of plus (+) for the comparison between aMCI and AD group and of asterisk (*) for the comparison between CN and AD groups.

The significance for data with italics emphasis is Bonferroni corrected *p* = 0.05.

#### 3.3.2 QSM

Compared to the CN, QSM values in the aMCI were significantly increased in the precuneus (*p* = 0.0255), allocortex (*p* = 0.0123), and anterior (*p* = 0.0316), and posterior (*p* = 0.0400) cingulate cortex. QSM values in the AD were significantly increased in hippocampus (*p* = 0.0029), amygdala (*p* = 0.0006), precuneus (*p* = 0.0003), thalamus (*p* = 0.0357), neocortex (*p* = 0.0001), allocortex (*p* = 0.0001), entorhinal cortex (*p* = 0.0038), and anterior (*p* = 0.0002) and posterior (*p* = 0.0010) cingulate cortex. Compared to the aMCI, QSM values in the AD were also significantly increased in the amygdala (*p* = 0.0490) and neocortex (*p* = 0.0267) (Table 3).

#### 3.3.3 ROC curve analyses

##### 3.3.3.1 Seven well-known iron accumulation ROIs

**Fig. 4:**
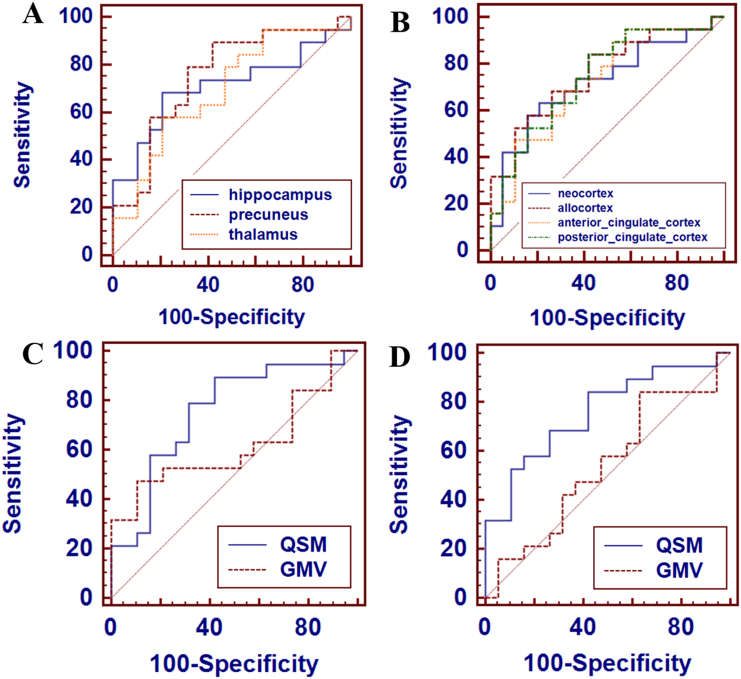
Results of ROC curve analysis of QSM values (A, B) obtained from the special region-of-interests (ROIs) to differentiate amnestic mild cognitive impairment (aMCI) subjects from cognitive normal (CN) subjects in the subjects and results of comparison of ROC curves (C, D) between QSM and GMV at the specific regions. (A) and (B) show the significant results of ROC curve analysis of QSM data in the well-known iron accumulation regions and the well-known amyloid β accumulation regions, respectively. (C) and (D) show the result of comparison of ROC curves to compare QSM and GMV in the precuneus and the allocortex regions, respectively.

Table 4 lists the results of ROC curves analysis of GMV and QSM values obtained from the seven well-known iron accumulation ROIs in the subjects with CN, aMCI, and AD. QSM values were differentiated between the CN and aMCI in the hippocampus (AUC = 0.709, *p* = 0.0189), precuneus (AUC = 0.753, *p* = 0.0020), and thalamus (AUC = 0.692, *p* = 0.0286). Fig. 4A summarizes the results of ROC curve analysis of QSM values obtained from the well-known iron accumulation regions. QSM values were differentiated between the aMCI and AD in only the amygdala (AUC = 0.739, *p* = 0.0044). QSM values were differentiated between the CN and AD in the hippocampus (AUC = 0.803, *p* < 0.0001), amygdala (AUC = 0.831, *p* < 0.0001), precuneus (AUC = 0.850, *p* < 0.0001), and thalamus (AUC = 0.742, *p* = 0.0036).

**Table 4:** Results of ROC curves analysis of GMVs and QSM values obtained from the seven well-known iron accumulation regions-of-interests (ROIs) in the subjects with Alzheimer's disease (AD), amnestic mild cognitive impairment (aMCI), and cognitively normal (CN) elderly.

ROI	Group		Statistics analysis results				
			SE [%]	SP [%]	Criterion	AUC	*p*
Hippocampus	CN vs aMCI	GMV	63.2	84.2	≤ 0.844	0.7922	*0.0001*
		QSM	68.4	78.9	>− 26.107	0.7091	*0.0189*
	aMCI vs AD	GMV	63.2	84.2	≤ 0.789	0.7340	*0.0047*
		QSM	57.9	68.4	>− 21.351	0.5927	0.3289
	CN vs AD	GMV	73.7	100.0	≤ 0.8186	0.9279	<* 0.0001*
		QSM	73.7	78.9	>− 26.107	0.8033	<* 0.0001*
Amygdala	CN vs aMCI	GMV	47.4	94.7	≤ 0.708	0.7368	*0.0038*
		QSM	68.4	63.2	>− 35.680	0.6288	0.1661
	aMCI vs AD	GMV	78.9	47.4	≤ 0.725	0.5872	0.3662
		QSM	89.5	57.9	>− 32.413	0.7396	*0.0044*
	CN vs AD	GMV	78.9	78.9	≤ 0.725	0.8171	<* 0.0001*
		QSM	94.7	68.4	>− 34.898	0.8310	<* 0.0001*
Globus pallibus	CN vs aMCI	GMV	57.9	63.2	≤ 0.121	0.5927	0.3276
		QSM	15.8	73.7	≤ 76.854	0.5235	0.8072
	aMCI vs AD	GMV	47.4	89.5	≤ 0.1132	0.5263	0.7957
		QSM	78.9	47.4	> 103.103	0.5678	0.4861
	CN vs AD	GMV	47.4	89.5	≤ 0.1132	0.5983	0.3203
		QSM	78.9	47.4	> 104.257	0.5346	0.7249
Precuneus	CN vs aMCI	GMV	47.4	89.5	> 0.4612	0.6177	0.2240
		QSM	59.5	57.9	>− 31.120	0.7534	*0.0020*
	MCI vs AD	GMV	84.2	52.6	≤ 0.458	0.6315	0.1614
		QSM	84.2	42.1	>− 26.961	0.6232	0.1881
	CN vs AD	GMV	26.3	89.5	≤ 0.432	0.5318	0.7432
		QSM	84.2	78.9	>− 26.955	0.8504	<* 0.0001*
Pulvinar	CN vs aMCI	GMV	63.2	73.7	≤ 0.351	0.6149	0.2312
		QSM	68.4	73.7	>− 11.925	0.6343	0.1609
	aMCI vs AD	GMV	78.9	57.9	≤ 0.349	0.6149	0.2336
		QSM	57.9	57.9	≤− 9.498	0.5069	0.9433
	CN vs AD	GMV	78.9	73.7	≤ 0.349	0.7285	*0.0083*
		QSM	63.2	73.7	>− 11.925	0.6398	0.1349
Putamen	CN vs aMCI	GMV	26.3	89.5	≤ 0.292	0.5457	0.6359
		QSM	78.9	63.2	> 9.770	0.6648	0.0748
	aMCI vs AD	GMV	73.7	68.4	≤ 0.298	0.6481	0.1114
		QSM	57.9	57.9	≤ 15.64	0.5290	0.7653
	CN vs AD	GMV	73.7	78.9	≤ 0.298	0.7368	*0.0053*
		QSM	73.7	63.2	> 9.770	0.6426	0.1241
Thalamus	CN vs aMCI	GMV	63.2	73.7	≤ 0.2582	0.6426	0.1332
		QSM	57.9	78.9	>− 25.775	0.6925	*0.0286*
	aMCI vs AD	GMV	89.5	31.6	> 0.2513	0.5429	0.6637
		QSM	68.4	57.9	>− 24.693	0.5678	0.4805
	CN vs AD	GMV	68.4	63.2	≤ 0.2598	0.6121	0.2344
		QSM	68.4	78.9	>− 25.775	0.7423	*0.0036*

Abbreviations: ROC, receiver operating characteristic; AUC, area under the ROC curve; GMV, gray matter volume; QSM, quantitative susceptibility mapping; SE, sensitivity; SP, specificity; *p*, *p*-value.

The significance for data with italics emphasis is Bonferroni corrected *p* = 0.05.

GMV values were differentiated between the CN and aMCI in the hippocampus (AUC = 0.792, *p* < 0.0001) and amygdala (AUC = 0.736, *p* = 0.0038). GMV values were differentiated between the aMCI and AD in only hippocampus (AUC = 0.734, *p* = 0.0047). GMV values were differentiated between the CN and AD in hippocampus (AUC = 0.927, *p* < 0.0001), amygdala (AUC = 0.817, *p* < 0.0001), pulvinar (AUC = 0.728, *p* = 0.0083), and putamen (AUC = 0.736, *p* = 0.0053). AUC values in the precuneus were greater in QSM (0.75346, *p* = 0.0020) than those in GMVs (0.61772, *p* = 0.2240) to differentiate aMCI from CN (Fig. 4C).

##### 3.3.3.2 Five amyloid β accumulation ROIs

ROC curves analysis of GMVs and QSM values obtained from the five amyloid β accumulation ROIs in the subjects with CN, aMCI, and AD are summarized in Table 5. QSM values were differentiated between CN and aMCI in the neocortex (AUC = 0.722, *p* = 0.0091), allocortex (AUC = 0.759, *p* = 0.0011), ACC (AUC = 0.728, *p* = 0.0063), PCC (AUC = 0.745, *p* = 0.0026). Fig. 4B shows the summary of the results of ROC curve analysis of QSM values obtained from the well-known amyloid β accumulation regions. QSM values were differentiated between the aMCI and AD in the neocortex (AUC = 0.717, *p* = 0.0115). QSM values were differentiated between the CN and AD in the neocortex (AUC = 0.858, *p* < 0.0001), allocortex (AUC = 0.889, *p* < 0.0001), entorhinal cortex (AUC = 0.731, *p* = 0.0050), ACC (AUC = 0.833, *p* < 0.0001), and PCC (AUC = 0.814, *p* < 0.0001). However, GMV values were only differentiated between CN and AD in the neocortex (AUC = 0.734, *p* = 0.0055). Fig. 4D shows that the area under curve (AUC) values in the allocortex were greater in QSM (0.7590, *p* = 0.0011) than those in GMVs (0.5373, *p* = 0.6997) to differentiate aMCI from CN.

**Table 5:** Results of ROC curves analysis of GMVs and QSM values obtained from the five amyloid β accumulation region-of-interests (ROIs) in the subjects with Alzheimer's disease (AD), amnestic mild cognitive impairment (aMCI), and cognitively normal (CN) elderly.

ROI	Group		Statistics analysis results				
			SE [%]	SP [%]	Criterion	AUC	*p*
Neocortex	CN vs aMCI	GMV	57.9	73.7	≤ 0.6737	0.5782	0.3682
		QSM	63.2	78.9	>− 35.944	0.7229	*0.0091*
	aMCI vs AD	GMV	57.9	78.9	≤ 0.6693	0.6620	*0.0751*
		QSM	57.9	89.5	>− 26.171	0.7174	*0.0115*
	CN vs AD	GMV	73.7	73.7	≤ 0.6737	0.7340	*0.0055*
		QSM	78.9	84.2	>− 35.166	0.8587	<* 0.0001*
Allocortex	CN vs aMCI	GMV	84.2	36.8	≤ 0.2365	0.5373	0.6997
		QSM	84.2	57.9	>− 29.417	0.7590	*0.0011*
	aMCI vs AD	GMV	78.9	68.4	≤ 0.2178	0.6565	0.1015
		QSM	78.9	47.4	>− 25.330	0.6288	0.1700
	CN vs AD	GMV	78.9	68.4	≤ 0.2178	0.6731	0.0605
		QSM	84.2	84.2	>− 25.537	0.8891	<* 0.0001*
Entorhinal cortex	CN vs aMCI	GMV	68.4	52.6	> 0.6213	0.5041	0.9663
		QSM	78.9	57.9	>− 28.979	0.6675	0.0668
	aMCI vs AD	GMV	57.9	57.9	≤ 0.6226	0.5124	0.8982
		QSM	52.6	78.9	>− 16.974	0.6011	0.2988
	CN vs AD	GMV	10.5	73.7	> 0.645	0.5013	0.9887
		QSM	52.6	89.5	>− 15.990	0.7313	*0.0050*
ACC	CN vs aMCI	GMV	31.6	94.7	> 0.6344	0.5152	0.8796
		QSM	68.4	68.4	>− 29.571	0.7285	*0.0063*
	aMCI vs AD	GMV	89.5	42.1	≤ 0.6276	0.5401	0.6868
		QSM	47.4	78.9	>− 21.466	0.6426	0.1209
	CN vs AD	GMV	47.4	68.4	≤ 0.6155	0.5457	0.6356
		QSM	89.5	63.2	>− 30.288	0.8337	<* 0.0001*
PCC	CN vs aMCI	GMV	57.9	57.9	> 0.587	0.5290	0.7637
		QSM	84.2	57.9	>− 31.363	0.7451	*0.0026*
	aMCI vs AD	GMV	57.9	89.5	≤ 0.5788	0.6315	0.1761
		QSM	52.6	68.4	>− 20.867	0.6011	0.2839
	CN vs AD	GMV	57.9	84.2	≤ 0.5788	0.6094	0.2600
		QSM	94.7	57.9	>− 31.363	0.8144	<* 0.0001*

Abbreviations: ACC, anterior cingulate cortex; PCC, posterior cingulate cortex; ROC, receiver operating characteristic; AUC, area under the ROC curve; GMV, gray matter volume; QSM, quantitative susceptibility mapping; SE, sensitivity; SP, specificity; *p*, *p*-value.

The significance for data with italics emphasis is Bonferroni corrected *p* = 0.05.

## 4 Discussion

### 4.1 Relationship between susceptibility effects and iron accumulation regions in the brain

QSM values were significantly increased from CN to AD in the hippocampus, amygdala, precuneus, and thalamus, indicating that the susceptibility-induced contents accumulated with disease progression. QSM values also differentiated aMCI from CN (Table 4). The precuneus was most sensitive to monitoring early changes. Measurement of GMV changes can be also used to differentiate groups. The hippocampus and amygdala differentiated the groups with QSM and GMV.

Iron levels at the cellular level are reported to be significantly elevated in neurofibrillary tangle using cytologic or immunocytochemical methods (Moretz et al., 1990). Our study is consistent with the suggestion of an iron overload in AD and supports the concept that iron homeostasis is disrupted in the AD brain. Presently, regional changes in iron accumulation were evident in brains of AD, except for the putamen. This means that in vivo measurement using QSM be a diagnostic tool for AD. It is still unclear whether iron accumulation is the cause or a consequence of the neurodegenerative cascade. For the present, this study provides important insights for the understanding of AD pathogenesis by monitoring the spatial distributions of iron deposition in cognitively normal and impaired individuals. Although QSM cannot distinguish which iron contribute to signals, the accumulation of iron from CN to AD in specific brain regions could help clarify the understanding of AD pathogenesis.

### 4.2 Relationship between susceptibility effects and the amyloid β protein in the brain

Brian regions displaying amyloid β accumulation in PET were significant susceptibility changes in AD, but GMV did not change (Table 3, Fig. 3). Furthermore, QSM values at the allocortex, ACC, and PCC were depicted in early changes of susceptibility in aMCI. However, GMV were not significantly different among the three groups in the predefined ROIs. Therefore, QSM may be much more sensitive than GMV to investigate group differences at those regions. While QSM values obtained from the neocortex, allocortex, ACC, and PCC were differentiated between CN and aMCI, GMV was not. This result may be explained by the early accumulation of amyloid β at those areas in the early stage of diseases without changing neuronal degeneration. This result explains that the amyloid imaging by PET is associated with susceptibility mapping by MRI.

One important feature of AD is associated with depositions of amyloid β peptide (Cuajungco et al., 2000, Hardy and Selkoe, 2002, Vandenberghe et al., 2013). The amyloid plaque overloads aggregate irons in brain tissue to induce chemical reduction of redox-inactive ferric irons to redox-active ferrous iron forms, producing oxidative stress and resultant neuronal damages (Everett et al., 2014). Because increased iron is closely related to greater production of amyloid β peptides, the assessment of brain iron levels has been a major hallmark of AD (Raven et al., 2013). One of the causes of the increased susceptibility value on AD tissue may be related to elevate iron and iron-mediated redox activity, even at preclinical and prodromal stages of AD (Smith et al., 2010). Neurofibrillary tangles develop beginning in the neocortical area (phase 1) and then spread to allocortical regions, such as the entorhinal cortex, and the anterior and posterior cingulate gyrus (phase 2) (Thal et al., 2002). Therefore, our results of QSM changes in these cortical lesions in AD and aMCI may indicate the contributions of both amyloid accumulation and neurofibrillary tangles. Presently, the susceptibility alterations were revealed to already start in the aMCI stage, which can be used as the early imaging marker. The QSM values can be distinguished CN from AD, as well as CN from aMCI groups. GMV may be classified between the CN and AD, but not between the CN and aMCI. Therefore, the QSM values in the precuneus and allocortex are more effective to investigate the early changes in the brain than GMV values. The quality of QSM data is depended on the optimization for the sequence parameters and post-processing algorithm. A clinical setting of QSM requires to shorten the scan time, reduce any artifacts, and to easily implement quantifications of the susceptibility. QSM still has a limitation to use for the individual clinical case to diagnosis iron uptake in a patient, indicating that QSM has to further develop to directly use as an imaging biomarker in individual patients.

## 5 Conclusions

The susceptibility difference in cognitive normal (CN), amnestic mild cognitive impairment (aMCI) and Alzheimer's disease (AD) elderly was more sensitive than gray matter volume (GMV) change in known regions of iron and the amyloid β accumulations, indicating the susceptibility changes caused by iron accumulation in the brain of Alzheimer's disease. Especially, QSM was better to differentiate between the CN and aMCI than GMV. Therefore, the QSM technology can be used as an auxiliary imaging factor for early diagnosis of AD. QSM can be used to evaluate the correlation with amyloid PET diagnosis.
